# Thermochemical Core–Shell Architecturing of Si‐SWCNT Composites for High‐Performance Li‐Ion Battery Anodes

**DOI:** 10.1002/advs.77441

**Published:** 2026-08-29

**Authors:** Dong Gyun Hong, Byeong Guk Kim, Jung‐Hyeok Park, Sunwoo Lee, Donghwi Cho, Jaeik Hyun, Ki‐Hun Nam, Youngoh Kim, Geon‐Woong Lee, Sunhye Yang, Seung Yol Jeong, Myungwoo Choi

**Affiliations:** ^1^ Nano Hybrid Technology Research Center Korea Electrotechnology Research Institute Changwon Republic of Korea; ^2^ Electric Energy Material Engineering University of Science and Technology Daejeon Republic of Korea; ^3^ Next Generation Battery Research Center Korea Electrotechnology Research Institute Changwon Republic of Korea; ^4^ School of Materials Science and Engineering Pusan National University Busan Republic of Korea; ^5^ Thin Film Materials Research Center Korea Research Institute of Chemical Technology Daejeon Republic of Korea; ^6^ Department of Materials Science and Engineering Korea University Seoul Republic of Korea; ^7^ Advanced Materials and Chemical Engineering University of Science and Technology Daejeon Republic of Korea; ^8^ Strategy and Policy Division Korea Electrotechnology Research Institute Changwon Republic of Korea

**Keywords:** anode, lithium‐ion battery, nanopore, silicon alloy, silicon carbide, single‐wall carbon nanotube

## Abstract

Silicon (Si) is a promising anode for next‐generation lithium‐ion batteries (LIBs) because of its ultrahigh theoretical capacity, yet its practical application is hindered by severe volume expansion, unstable interfaces, and sluggish reaction kinetics. Here, we report a thermochemically engineered core–shell Si architecture integrating alloy stabilization, nanoporous strain buffering, and chemically anchored conductive networks. Starting from a SWCNT‐coated Mg_2_Si precursor, a CH_4_‐assisted thermochemical treatment induces localized exothermic Mg oxidation, followed by selective MgO etching to construct a SiC‐anchored SWCNT conductive network and a nanoporous Si shell surrounding a robust Mg‐containing Si core. Reactive molecular dynamics simulations reveal that localized hot‐atom generation during Mg oxidation activates CH_4_ molecules, providing atomistic insight into the low‐temperature formation of the SiC interfacial layer. The resulting architecture decouples mechanical strain from charge transport, enabling an initial reversible capacity of 1890 mAh g^−1^ with 74% capacity retention after 100 cycles. Graphite composite electrodes containing 20 wt.% Si deliver 640 mAh g^−1^ and retain 78% capacity after 200 cycles, substantially exceeding the Si content used in commercial electrodes. Pouch‐type full cells paired with Li[Ni_0.6_Co_0.2_Mn_0.2_]O_2_ cathodes achieve an energy density of 275 Wh kg^−1^ with 75% capacity retention after 1000 cycles, demonstrating a scalable strategy for high‐energy‐density LIBs.

## Graphical abstract

A thermochemically engineered core–shell Si architecture is developed through the controlled evolution of a SWCNT‐coated Mg_2_Si precursor. The process simultaneously generates a nanoporous Si shell for strain accommodation and *Li* ^+^ transport, a chemically anchored SiC interfacial layer for robust mechanical and electrical coupling, and a Mg‐containing Si core for structural stabilization. This spatially differentiated design effectively mitigates the intrinsic challenges of Si anodes, including severe volume expansion and interfacial degradation. As a result, the nanoporous Si/SiC/SWCNT anode enables stable operation at high Si loadings and delivers outstanding performance in graphite‐composite electrodes and pouch‐type full cells, demonstrating a practical pathway toward high‐energy‐density lithium‐ion batteries.

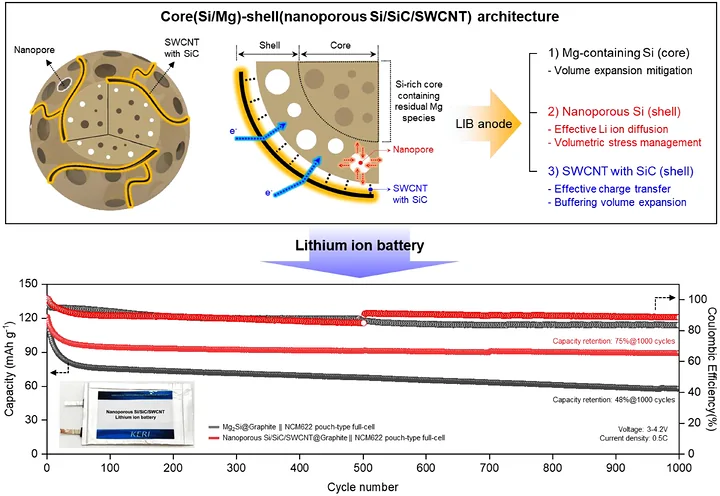

## 1 Introduction

The accelerating demand for high‐energy‐density lithium‐ion batteries (LIBs), driven by electrified transportation and large‐scale energy storage systems, has positioned silicon (Si) as one of the most promising alternatives to conventional graphite anodes. Si offers an exceptional theoretical specific capacity of 3579 mAh g^−1^ (Li_15_Si_4_), nearly an order of magnitude higher than that of graphite (372 mAh g^−1^), along with a relatively low lithiation potential (∼0.4 V vs. *Li*/*Li* ^+^), enabling significantly higher energy densities [1]. Despite these advantages, the practical implementation of Si anodes remains fundamentally constrained by severe structural and interfacial instabilities during electrochemical cycling. The most critical challenge arises from the drastic volumetric expansion of Si (>300%) upon lithiation, which induces particle pulverization, loss of electrical contact, and continuous reconstruction of the solid–electrolyte interphase (SEI) [2, 3, 4]. These coupled degradation processes ultimately lead to rapid capacity decay and poor cycling stability. In addition, the intrinsically low electrical conductivity of Si (10^−5^–10^−3^ S cm^−1^), together with its sluggish *Li* ^+^ diffusion kinetics (10^−14^–10^−13^ cm^2^ s^−1^), further exacerbates the reaction polarization and limits rate capability in practical electrodes [5].

To overcome these intrinsic mechanical and electrochemical hurdles, research has converged on four primary paradigms: metallic alloying [6, 7], nanocarbon integration [8, 9], porosity engineering [11, 12, 13, 14], and interfacial stabilization [15, 16, 17]. First, metallic alloying involves embedding Si within electrochemically inactive or semi‐active matrices (e.g., Mg, Fe, Ni) [18, 19]. This metallic phase serves as a structural backbone that effectively buffers absolute volume fluctuations and homogenizes internal stress distribution, thereby preventing the macroscopic fracture of the active material [20, 21]. Second, the integration of conductive nanocarbon networks, specifically single‐walled carbon nanotubes (SWCNTs) or graphene, is employed to establish a resilient, percolative conductive framework [22]. Unlike simple carbon coatings, these high‐aspect‐ratio nanocarbons maintain long‐range electrical connectivity even as the Si particles undergo repeated expansion and contraction [23]. Third, porosity engineering has been extensively explored to introduce internal void spaces capable of accommodating lithiation‐induced expansion [24]. By introducing a nanoporous structure—typically optimized at 40%–50% porosity—the effective volumetric expansion can be theoretically confined to below 100%, significantly alleviating the strain on the surrounding electrode environment. Finally, interfacial stabilization through the formation of robust layers, such as silicon carbide (SiC) or crystalline carbon shells, is critical to suppress parasitic electrolyte decomposition and ensure mechanical coupling between Si and the conductive additives [25].

Despite the considerable progress achieved through these individual strategies, none of them alone can fully resolve the intrinsic limitations of Si anodes due to their inherent trade‐offs. For example, porosity engineering effectively mitigates mechanical stress by introducing internal void space; however, the accompanying increase in surface area accelerates electrolyte decomposition and promotes excessive SEI formation, often leading to significant losses in initial Coulombic efficiency (ICE) [26]. Similarly, conductive nanocarbon integration improves electronic transport and structural flexibility but often relies on weak physical contacts that are susceptible to interfacial degradation during repeated volume changes. Strengthening the Si–carbon interface through chemically anchored layers such as SiC or graphitic shells can improve mechanical coupling and interfacial stability, yet these approaches typically require extreme processing temperatures exceeding 1000°C [27], which may induce undesirable Si grain growth or degrade the integrity of nanocarbon networks. Consequently, an ideal Si anode architecture must simultaneously integrate multiple design strategies—such as alloy stabilization, conductive nanocarbon networks, nanoporous strain buffering, and interfacial engineering—within a single material system. Developing a scalable synthetic route capable of orchestrating these complementary strategies under moderate processing conditions therefore remains a critical challenge for practical Si anode manufacturing [28, 29].

Here, we report a thermochemically engineered Si anode architecture that resolves these competing requirements through a controlled structural evolution pathway. By utilizing a mechanically alloyed Mg_2_Si precursor coated with SWCNTs, we induce a selective oxidation of Mg, which serves as a dual‐function structural driver. First, the exothermic transformation of Mg into MgO generates localized thermal energy that promotes the in situ formation of a robust SiC interfacial layer between Si and SWCNTs at a relatively moderate temperature (∼650°C). Reactive molecular dynamics simulations further reveal the atomistic origin of this process, revealing that localized hot‐atom generation during Mg oxidation activates CH_4_ molecules and initiates interfacial Si─C bond formation under moderate thermal conditions. Second, the resulting MgO acts as an intrinsic sacrificial template that, upon selective acid etching, yields a spatially confined nanoporous shell while preserving a dense and mechanically stable Mg‐containing Si core. This spatially differentiated architecture—comprising a nonporous core that maintains structural integrity and a nanoporous Si/SiC/SWCNT shell that facilitates rapid ion/electron transport—effectively decouples electrochemical kinetics from mechanical strain accommodation. As a result, the composite enables a high Si‐loading electrode configuration. Even when blended with commercial graphite at a weight ratio of 2:8 (20 wt.% Si composite), the electrode maintains remarkable electrochemical stability. Full‐cell evaluations paired with high‐voltage Li[Ni_0.6_Co_0.2_Mn_0.2_]O_2_ (NCM622) cathodes deliver a reversible capacity of 128 mAh g^−1^ with a capacity retention of 75% over 1000 cycles at a current density of 0.5C. These results demonstrate a scalable thermochemical pathway toward next‐generation high‐capacity Si‐based anodes by synergistically integrating alloying, nanocarbon hybridization, nanoporous engineering, and interfacial stabilization within an energetically efficient synthesis framework.

## 2 Results and Discussion

### 2.1 Thermochemically Driven Synthesis of Core–Shell Nanoporous Si/SiC/SWCNT Hybrid Architecture

**FIGURE 1:**
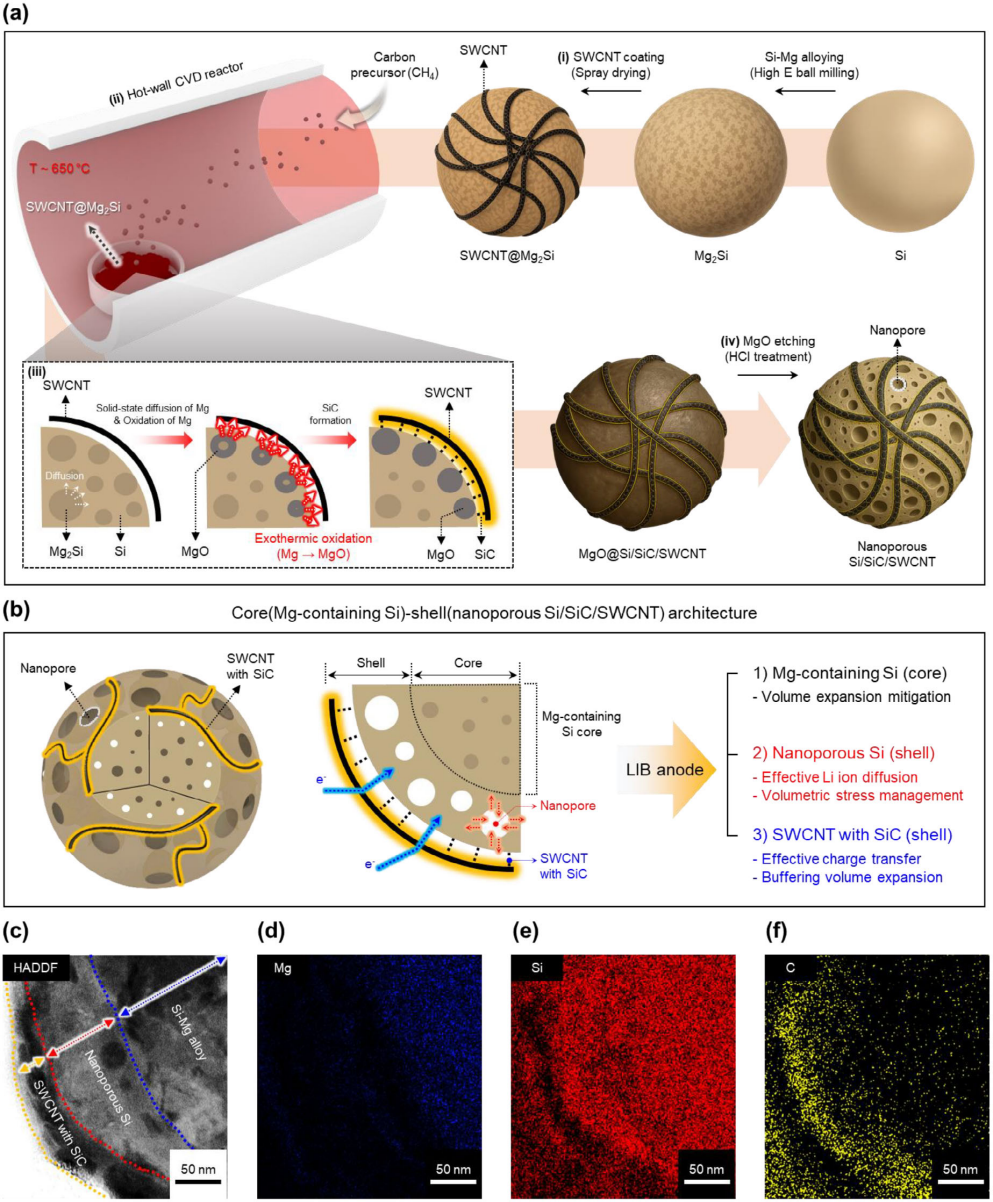
Synthesis and structural characterization of the core–shell nanoporous Si/SiC/SWCNT hybrid. (a) Schematic illustration of the fabrication process for the nanoporous Si/SiC/SWCNT hybrid: (i) SWCNT coating on Mg_2_Si, (ii) CH_4_‐based heat treatment in a hot‐wall CVD chamber, (iii) thermally induced SiC interface formation, and (iv) selective MgO etching for nanopore generation. (b) Conceptual illustration of the core(Mg‐containing Si core)–shell(nanoporous Si/SiC/SWCNT) architecture and its synergistic advantages for LiB anodes, including accelerated *Li* ^+^ diffusion and effective strain mitigation. (c) Cross‐sectional HAADF‐TEM image of the nanoporous Si/SiC/SWCNT hybrid, highlighting the distinct interfacial layers. (d–f) Corresponding EDS elemental maps for (d) Mg, (e) Si, and (f) C, demonstrating the spatial distribution of the core–shell components. Scale bars, 50 nm.

To overcome the inherent kinetic and mechanical limitations of Si anodes, we developed a spatially differentiated core(Mg‐containing Si core)–shell(nanoporous Si/SiC/SWCNT) hybrid architecture via a controlled thermochemical evolution pathway (Figure 1a). The synthesis begins with the preparation of Mg_2_Si microparticles via high‐energy ball milling (HEBM) [30, 31, 32], followed by conformal encapsulation with a percolative network of SWCNT through a spray‐drying process (Figure 1a, (i)). This hybrid precursor provides both the alloy framework and the conductive carbon scaffold required for the subsequent structural transformation. The key structural evolution is initiated during thermal treatment at ∼650°C under an Ar/CH_4_ atmosphere in a hot‐wall chemical vapor deposition (CVD) reactor (Figure 1a, (ii)). During this process, Mg atoms within the Mg_2_Si phase undergo solid‐state diffusion toward the particle surface, where thermodynamically favorable oxidation leads to the formation of MgO. This oxidation process is highly exothermic $\left(\Delta {H_{f}}^{\circ }=601.7\;\mathrm{kJ}\;\mathrm{mo}l^{-1}\right.$), generating localized heat that significantly exceeds the nominal furnace temperature. Such localized thermal activation promotes the interfacial reaction between Si and the SWCNT framework, leading to the in situ formation of a chemically robust SiC interfacial layer (Figure 1a, (iii)). Unlike conventional SiC synthesis—which typically requires temperatures exceeding 1500°C [33] —this thermochemically assisted route enables the formation of SiC at a substantially lower processing temperature by exploiting the localized heat released during Mg oxidation. In addition to driving the SiC interfacial reaction, the Mg oxidation step simultaneously produces MgO that is spatially distributed within the outer region of the particle. Subsequent selective acid etching removes the MgO phase, generating a hierarchical nanoporous Si shell surrounding the Mg‐containing Si core (Figure 1a, (iv)). The resulting porosity provides internal void space that can effectively accommodate the large volumetric expansion of Si during lithiation.

As schematically illustrated in Figure 1b, the final architecture exhibits a functionally differentiated core–shell configuration that synergistically addresses both mechanical stability and electrochemical kinetics. The interior Mg‐containing Si core serves as a mechanically robust, semi‐active backbone that partially participates in lithiation while helping to mitigate the overall volume fluctuation of the particle. In contrast, the outer shell integrates three critical functional elements: (i) nanopores that buffer lithiation‐induced strain and shorten *Li* ^+^ diffusion pathways, (ii) a SiC interfacial layer that chemically anchors the Si framework to the carbon network, and (iii) an interconnected SWCNT scaffold that provides long‐range electronic conductivity. To experimentally verify this structural evolution, cross‐sectional high‐angle annular dark‐field transmission electron microscopy (HAADF‐TEM) and energy‐dispersive X‐ray spectroscopy (EDS) mapping were conducted (Figure 1c–f). The HAADF‐TEM image clearly reveals the distinct core–shell morphology, where a nanoporous Si region with a thickness of approximately 50–100 nm forms the outer shell and is intimately integrated with the SWCNT/SiC network (Figure 1c). Corresponding elemental maps further confirm the spatial separation of phases: Mg signals are predominantly localized within the dense interior region, indicating the retention of the Mg‐containing Si core (Figure 1d), whereas Si and C signals are co‐distributed along the outer shell, consistent with the formation of the SiC/SWCNT hybrid interface (Figure 1e,f). This spatially differentiated architecture effectively decouples electrochemical reaction sites from mechanical strain accumulation, which is expected to enable both rapid reaction kinetics and enhanced cycling stability.

### 2.2 Physicochemical Characterization of Core–Shell Nanoporous Si/SiC/SWCNT Hybrids

**FIGURE 2:**
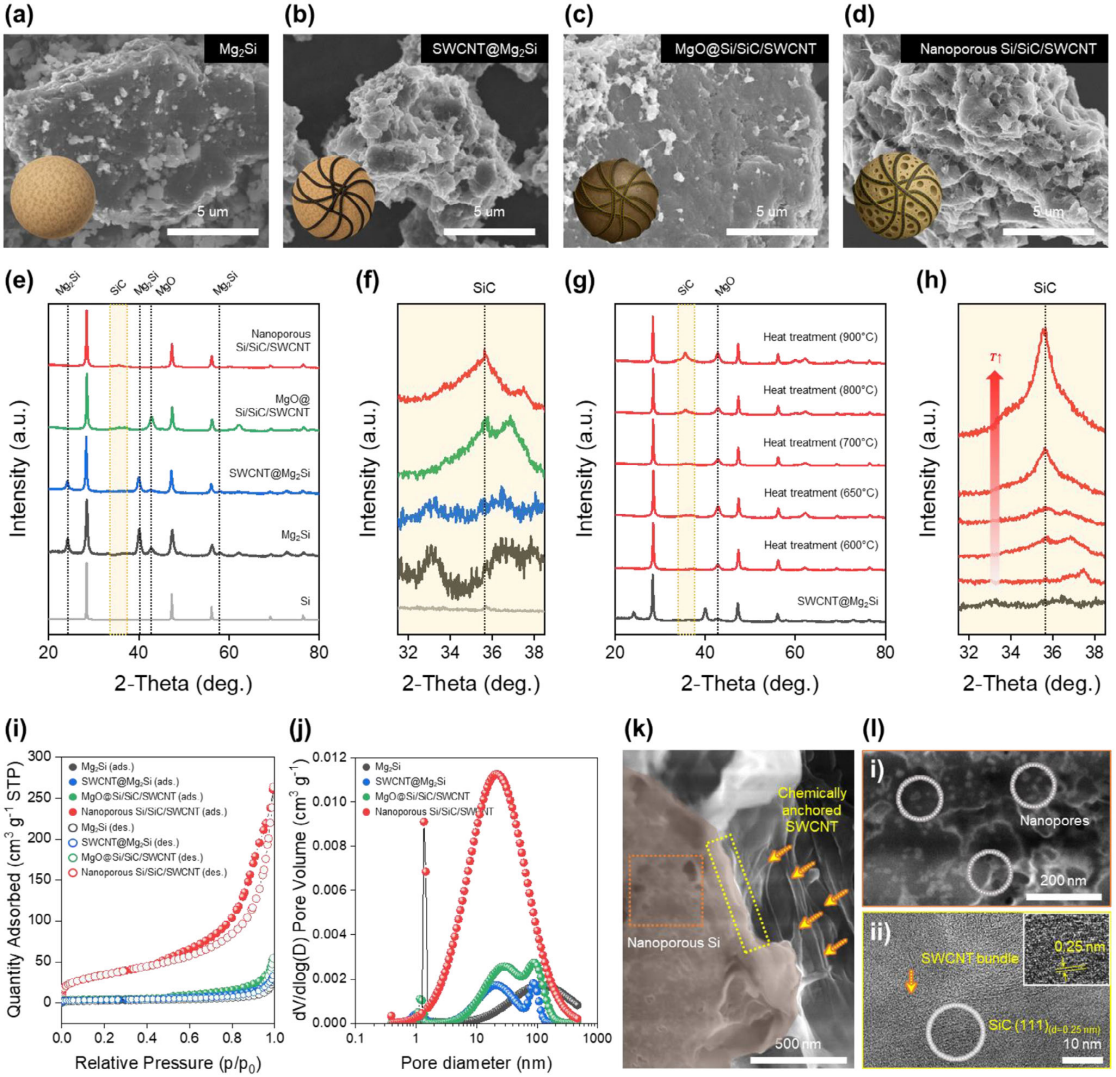
Morphological and structural evolution of the nanoporous Si/SiC/SWCNT hybrid. (a–d) SEM images and corresponding schematics of (a) Mg_2_Si, (b) SWCNT@Mg_2_Si, (c) MgO@Si/SiC/SWCNT, and (d) nanoporous Si/SiC/SWCNT, showing the surface morphological changes during the synthesis steps. (e) XRD patterns of the samples. (f) provides a magnified view of the dashed box in (e). (g) Temperature‐dependent XRD patterns of the samples synthesized under a CH_4_ atmosphere from 600°C to 900°C. (h) demonstrates the evolution of the SiC phase with increasing temperature. (i,j) Nitrogen adsorption–desorption isotherms and corresponding pore size distribution curves. The pore size distribution of Mg_2_Si is nearly indistinguishable because of its negligible pore volume. (k) FIB‐assisted cross‐sectional SEM image of the nanoporous Si/SiC/SWCNT, displaying the core‐shell‐like integrated structure. (l)‐i), Magnified SEM image of the orange dashed box in (k) clearly resolving the distributed nanopores within the Si shell. (l)‐ii), High‐resolution TEM image of the yellow dashed box in (k), confirming the crystalline SiC interface (*d*_(111)_ = 0.25 nm) chemically bonded with the SWCNT bundles.

The systematic structural evolution from the Mg_2_Si precursor to the nanoporous Si/SiC/SWCNT hybrid was comprehensively investigated through multiscale imaging and spectroscopic analysis. Scanning electron microscopy (SEM) images clearly illustrate the stepwise morphological evolution during the thermochemical synthesis (Figure 2a–d). The pristine Mg_2_Si precursor produced by HEBM consists of relatively dense microparticles with irregular surfaces, reflecting the severe plastic deformation and particle fracture induced during the mechanical alloying process (Figure 2a). After the spray‐drying step, the particles become uniformly wrapped by an interconnected network of SWCNTs, forming the SWCNT@Mg_2_Si hybrid precursor (Figure 2b). The pristine SWCNTs exhibited an average tube diameter of approximately 1.32 nm, estimated from the radial breathing mode (RBM) Raman peak using the relation ω_RBM_ = 223.5/*d*_t_+12.5, where ω_RBM_ is the RBM frequency (cm^−1^) and *d*_t_ is the nanotube diameter (nm) (Figure S1a,b), and a typical tube length of ≥5 µm according to the manufacturer's specification (OCSiAl TUBALL). This conductive carbon network serves as an electrically conductive scaffold and a mechanically compliant framework for the subsequent interfacial SiC formation. Following the CH_4_‐assisted thermal treatment, a significant morphological transformation occurs (Figure 2c). The particle surface becomes noticeably rough and textured, which is attributed to the formation of MgO within the outer region of the particles during the thermochemical evolution. This intermediate structure, denoted as MgO@Si/SiC/SWCNT, consists of MgO species distributed within the shell region together with the newly formed SiC/SWCNT framework. Upon subsequent selective acid etching, the MgO phase is removed, leaving behind a well‐developed hierarchical nanoporous structure (Figure 2d). The resulting nanoporous Si/SiC/SWCNT hybrid exhibits a highly roughened surface with interconnected pore channels, indicating that the MgO phase acts as an effective sacrificial template for generating the porous shell architecture.

The corresponding phase evolution was examined by X‐ray diffraction (XRD), Raman spectroscopy, and X‐ray photoelectron spectroscopy (XPS). Weak MgO diffraction peaks are already detectable in the Mg_2_Si precursor, which are attributed to slight surface oxidation of Mg during post‐milling handling (Figure 2e). After the thermal treatment, however, the MgO peak intensity increases markedly, indicating further oxidation of the remaining Mg during the thermochemical process (Figure 2e). To identify the oxygen source responsible for this oxidation, thermogravimetric analysis (TGA) was performed on the commercial SWCNT paste. The TGA results reveal that the paste contains 66.19 wt.% sodium carboxymethyl cellulose (SCMC), which releases oxygen‐containing species during thermal decomposition (Figure S1c). A control experiment using graphite without oxygen‐containing polymeric species produced neither MgO nor SiC diffraction peaks after identical heat treatment (Figure S2), confirming that oxygen released from SCMC is responsible for Mg oxidation and the subsequent formation of the SiC interfacial layer. Consequently, the XRD patterns verify the successful conversion of Mg_2_Si into crystalline Si together with the formation of the characteristic β‐SiC (111) phase at ∼35.6° (Figure 2f). Concurrently, Raman spectra across all synthesis stages exhibit prominent G‐band (∼1590 cm^−^^1^) and 2D‐band (∼2680 cm^−^^1^) signals, indicating that the SWCNT conductive network remains structurally robust throughout the thermochemical processing (Figure S3a). The evolution of the SiC interfacial layer was further investigated by varying the thermal‐treatment temperature. Temperature‐dependent XRD measurements reveal a progressive increase in the SiC (111) diffraction peak from 600°C to 900°C under an Ar/CH_4_ atmosphere (Figure 2g,h), indicating continuous growth of the interfacial SiC phase. XPS analysis further confirms the chemical integration between Si and SWCNTs. Unlike the physically mixed precursor, both the intermediate and final hybrid samples exhibit distinct Si─C bonding states in the Si 2p (∼100.5 eV) and C 1s (∼282.6 eV) spectra (Figure S3b,c), confirming the formation of a chemically anchored SiC interface.

To further understand the structural evolution during thermochemical processing, the MgO@Si/SiC/SWCNT intermediate prior to acid etching was examined. As illustrated in the proposed mechanism (Figure S4a), thermal treatment at ∼650 °C initiates the outward solid‐state diffusion of Mg from the Mg_2_Si‐derived core. Cross‐sectional TEM imaging combined with EDS elemental mapping confirms that Mg remains localized within the particle core (Figure S4b–f), whereas the corresponding XRD patterns no longer exhibit crystalline Mg_2_Si diffraction peaks (Figure 2e), suggesting that the remaining Mg exists as amorphous or XRD‐undetectable Mg‐containing species. Meanwhile, the outward‐diffusing Mg species undergo oxidation to form MgO, which becomes spatially distributed within the outer region of the particle. This localized reaction environment is particularly important, as the exothermic oxidation of Mg generates localized thermal energy that facilitates the interfacial reaction between Si and the carbon network, thereby enabling SiC formation under relatively moderate external temperatures.

The resulting structural evolution ultimately produces a well‐defined mesoporous architecture. Nitrogen adsorption–desorption isotherms reveal a systematic evolution in porosity across the synthesis stages (Figure 2i). The Mg_2_Si precursor, SWCNT@Mg_2_Si, and MgO@Si/SiC/SWCNT intermediate exhibit relatively low nitrogen uptake with specific surface areas (SSAs) of 10.52, 12.96, and 14.51 m^2^ g^−1^, respectively, indicating that a well‐developed pore network has not yet formed. In contrast, the final nanoporous Si/SiC/SWCNT hybrid exhibits a typical type IV adsorption–desorption isotherm with a pronounced H3‐type hysteresis loop, characteristic of an interconnected mesoporous structure [34]. Consequently, its SSA increases significantly to 126.83 m^2^ g^−1^ after selective removal of the MgO sacrificial template. The corresponding pore size distribution is centered at 43.57 nm, confirming the formation of well‐defined mesopores (Figure 2j).

Finally, the hierarchical core–shell architecture was directly visualized by Focused ion beam (FIB)‐assisted cross‐sectional SEM and high‐resolution TEM. FIB‐assisted cross‐sectional SEM images reveal a nanoporous Si shell intimately integrated with the SWCNT conductive framework (Figure 2k,l‐(i)). High‐resolution TEM further provides atomic‐scale evidence of the interface (Figure 2l‐(ii)). The observed lattice fringes with a d‐spacing of ∼0.25 nm correspond to crystalline SiC (111) planes that are directly grafted onto the SWCNT bundles. Such hierarchical structural integration—from atomic‐scale interfacial bonding to mesoscale buffering pores—is expected to provide both mechanical resilience and efficient charge transport during electrochemical cycling.

### 2.3 Atomistic Mechanism of Thermochemically Induced Si─C Bond Formation

**FIGURE 3:**
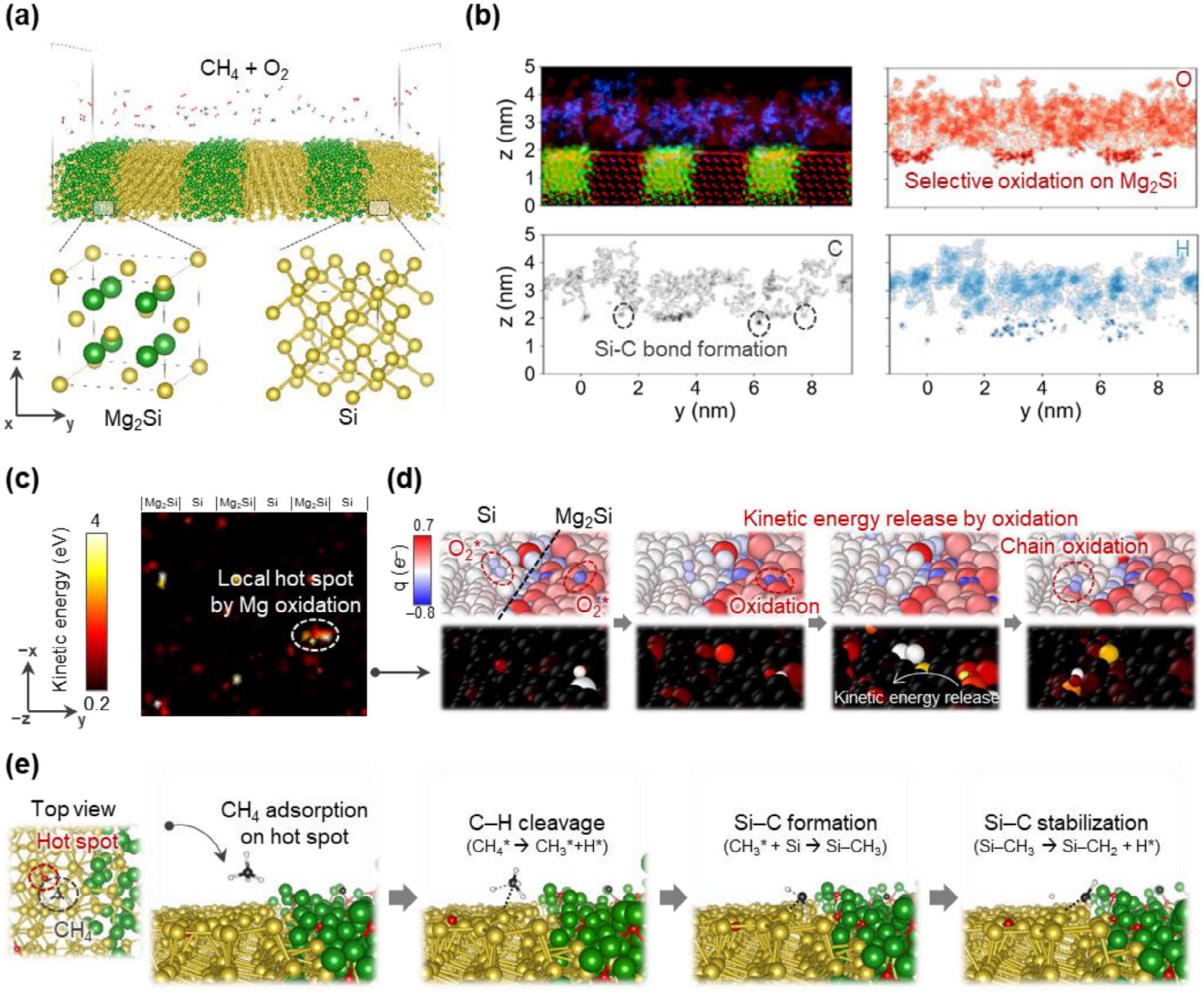
Reactive molecular dynamics simulations revealing oxidation‐induced hot‐atom‐assisted Si─C bond formation. (a) Atomistic model of the Mg_2_Si/Si heterogeneous surface exposed to a CH_4_/O_2_ atmosphere. (b) Atomic distributions during oxidation process. In the overlay, Si, Mg, C, H, and O atoms are shown in red, green, cyan, blue, and dark red, respectively. (c) Spatial distribution of atomic kinetic energy, revealing oxidation‐induced localized hot spots generated by the exothermic oxidation of Mg. (d) Representative reaction trajectory illustrating O_2_ dissociation, Mg oxidation, localized chemical to kinetic energy conversion, and the transient hot‐atom state responsible for rapid surface diffusion. (e) Proposed mechanism of hot‐atom‐assisted methane activation, where CH_4_ preferentially adsorbs at oxidation‐induced hot spots, undergoes C─H bond cleavage, forms Si─C bonds, and is followed by structural rearrangement through hydrogen incorporation into the Si lattice.

To gain atomistic insight into the low‐temperature formation of the SiC interfacial layer, reactive molecular dynamics (rMD) simulations were performed to directly investigate the local reaction processes occurring during the thermochemical treatment (Figure 3). In particular, the simulations were designed to clarify whether the exothermic oxidation of Mg could create localized reaction environments capable of activating otherwise inert carbon species and promoting Si─C bond formation under moderate external heating conditions. A heterogeneous model consisting of periodically arranged Mg_2_Si alloy and pure Si domains was constructed and exposed to a mixed CH_4_/O_2_ atmosphere, representing the carbon‐ and oxygen‐containing species present during the thermochemical process (Figure 3a). Such a heterogeneous configuration enables direct comparison between Mg‐containing and Mg‐free Si regions under identical reaction conditions while eliminating differences arising from the surrounding atmosphere.

The atomistic evolution clearly demonstrates that oxidation occurs preferentially on the Mg_2_Si domains, whereas the neighboring Si domains remain largely unreactive despite continuous exposure to both CH_4_ and O_2_ (Figure 3b). This selective oxidation behavior originates from the strong thermodynamic driving force for Mg oxidation and results in the rapid conversion of chemical energy into atomic kinetic energy. Consequently, highly localized high‐energy hot spots are generated around the oxidation sites, producing transient nonequilibrium atomic motion that is significantly more energetic than the surrounding lattice (Figure 3c). Rather than remaining localized, these hot atoms continuously transfer kinetic energy into adjacent Si atoms through atomic collisions, allowing the transient high‐energy states to propagate beyond the oxidized Mg_2_Si region (Figure 3d). Such localized energy transfer creates dynamic reaction sites that cannot be described solely by the nominal furnace temperature, providing an atomistic explanation for the unique reaction environment generated during the thermochemical process.

The simulations further reveal that these oxidation‐induced hot spots play a decisive role in activating CH_4_ molecules. When CH_4_ adsorbs onto the locally excited Si surface (CH_4_*), the enhanced atomic vibration substantially weakens the C─H bonds, leading to spontaneous bond dissociation without requiring additional external thermal activation (Figure 3e). The resulting CH_3_ radicals subsequently react with neighboring Si atoms to form stable Si─C bonds, while the remaining hydrogen atoms diffuse into the Si lattice during structural relaxation. In contrast, on Si regions that are not activated by the oxidation‐induced hot‐atom behavior, neither C─H bond cleavage nor Si─C bond formation is observed throughout the simulation despite identical CH_4_/O_2_ exposure conditions. These results indicate that the carbon precursor alone is insufficient to induce Si─C bond formation and that localized energy generated by Mg oxidation is the critical factor governing the interfacial reaction.

These atomistic observations provide direct mechanistic support for the experimentally observed formation of the SiC interfacial layer during thermochemical treatment. Rather than uniformly heating the entire particle, the exothermic oxidation of Mg generates transient localized reaction sites that activate otherwise inert CH_4_ molecules through hot‐atom‐mediated energy transfer, thereby enabling Si─C bond formation under a moderate furnace temperature of approximately 650°C. Collectively, these simulations are in excellent agreement with the experimental observations, providing atomistic insight into how localized Mg oxidation activates CH_4_ molecules and promotes the thermochemical formation of the SiC interfacial layer under moderate thermal conditions.

### 2.4 Superior Electrochemical Performance and Lithium Storage Kinetics of the Core–Shell Nanoporous Si/SiC/SWCNT Hybrids

To systematically evaluate the electrochemical advantages of our core‐shell hybrid architecture, we first established the significance of the precursor synthesis. The in‐house synthesized Mg_2_Si precursor via HEBM of elemental Si and Mg powders was compared with commercially available bulk Mg_2_Si. As demonstrated in Figure S5, the HEBM‐derived precursor yielded a significantly higher initial discharge capacity (∼1477 mAh g^−1^), a superior initial Coulombic efficiency (ICE, ∼84%), and improved capacity retention (55%@100 cycles) relative to the commercial counterpart (∼645 mAh g^−1^, ∼76%, and 37%@100 cycles respectively). To decouple the influence of particle size, commercial Mg_2_Si was further subjected to the same HEBM process. Although particle‐size refinement improved its electrochemical performance, the ball‐milled commercial Mg_2_Si remained markedly inferior to the HEBM‐derived Mg_2_Si precursor (Figure S6), highlighting the critical role of the optimized Si/Mg compositional ratio. Furthermore, electrochemical comparison of Mg_2_Si alloys with different Si/Mg ratios revealed that neither Si‐rich nor Mg‐rich compositions provide the optimal electrochemical performance. Instead, an intermediate Si/Mg ratio achieves the best balance between reversible capacity and cycling stability (Figure S7). These results demonstrate that the superior electrochemical performance originates from the combined effects of particle‐size refinement and compositional optimization achieved through the HEBM process.

Prior to evaluating electrochemical properties, the intrinsic electrical properties of the fabricated electrodes were analyzed to validate the effectiveness of the synergistic effects of the integrated core–shell architecture. Quantitative resistance analysis reveals that the nanoporous Si/SiC/SWCNT electrode exhibits the lowest active material and interface resistances among the four comparison groups (Figure S8). Although SiC itself is not a conductive phase, the chemically bonded SiC interphase effectively strengthens the interfacial interaction between Si and the interconnected SWCNT network, thereby stabilizing electrical contact and suppressing contact degradation within the conductive framework [35]. Combined with the continuous electron transport pathways provided by SWCNTs and the increased effective contact area afforded by the nanoporous architecture, this hierarchical core–shell design establishes an efficient percolative conductive framework that minimizes both the active material resistance and the interface resistance.

**FIGURE 4:**
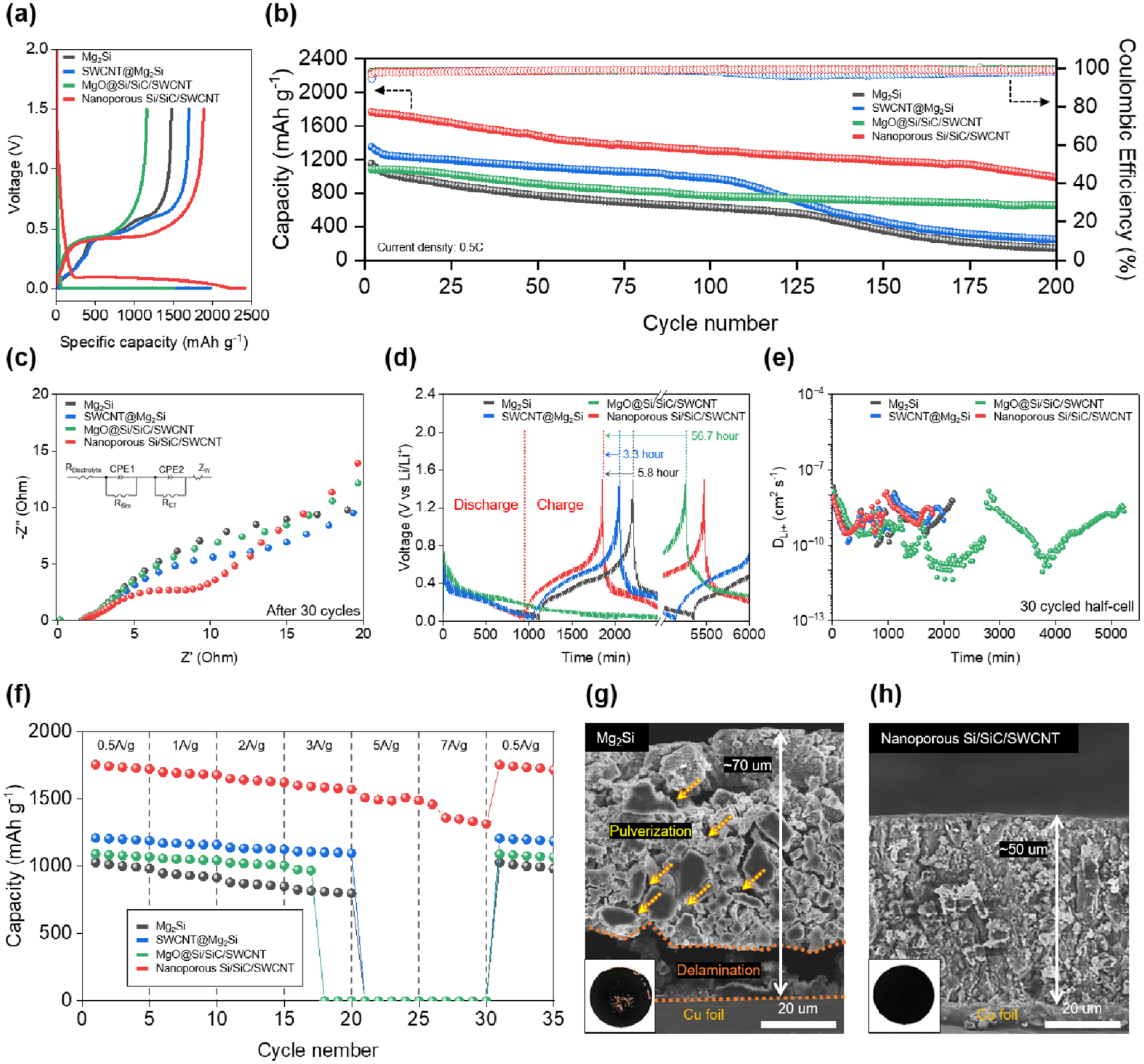
Electrochemical performance and kinetic analysis of nanoporous Si/SiC/SWCNT anodes. (a) Galvanostatic charge/discharge profiles of Mg_2_Si, SWCNT@Mg_2_Si, MgO@Si/SiC/SWCNT, and nanoporous Si/SiC/SWCNT electrodes at a constant current density. (b) Comparative cycling performance and corresponding Coulombic efficiencies over extended cycles. (c) Nyquist plots obtained from EIS measurements after 30 cycles. (d) Potentiostatic response during the GITT for kinetic evaluation. (e) *D*(*Li* ^+^) calculated from the GITT data as a function of state of charge. (f) Rate capability at various current densities ranging from 0.5 to 7 A g^−1^. (g,h) Cross‐sectional SEM images of the Mg_2_Si and nanoporous Si/SiC/SWCNT electrodes, respectively, after 100 electrochemical cycles. All electrode cross‐sections were prepared using the identical ion‐milling procedure under the same experimental conditions to ensure a direct comparison. The insets show digital photographs of the corresponding electrodes, highlighting their macroscopic structural integrity.

Building upon these optimized structural and electrical foundations, the nanoporous Si/SiC/SWCNT hybrid demonstrates outstanding *Li* ^+^ storage performance. The initial galvanostatic charge/discharge profile delivers a highly reversible capacity of 1890 mAh g^−1^ at a current density of 0.5 A g^−1^ (Figure 4a). More importantly, the nanoporous Si/SiC/SWCNT hybrid anode exhibits exceptional long‐term cycling stability, retaining a discharge capacity of 1000 mAh g^−1^ after 200 cycles (Figure 4b and Figure S9). The intermediate electrodes exhibit progressively improved electrochemical performance as the thermochemical evolution proceeds. The pristine Mg_2_Si electrode rapidly loses capacity (145 mAh g^−1^ after 200 cycles) because of its poor electrical conductivity and severe structural degradation during repeated lithiation/delithiation. Incorporation of the SWCNT network moderately improves cycling stability (251 mAh g^−1^ after 200 cycles) by providing additional electron transport pathways; however, the physically attached SWCNTs cannot effectively maintain electrical contact as the active material undergoes repeated volume changes. Following thermochemical treatment, the MgO@Si/SiC/SWCNT intermediate exhibits substantially improved capacity retention (656 mAh g^−1^ after 200 cycles), demonstrating the beneficial effect of the chemically integrated SiC/SWCNT conductive framework. Nevertheless, the retained MgO phase partially blocks *Li* ^+^ transport and reduces active material utilization. After selective removal of MgO, the resulting nanoporous Si/SiC/SWCNT architecture simultaneously provides efficient *Li* ^+^ diffusion through the nanoporous shell, robust electrical connectivity through the SiC‐anchored SWCNT network, and mechanical stabilization by the retained Mg‐containing Si core, thereby delivering the best overall electrochemical performance among all comparison groups.

The superior electrochemical performance is further supported by cyclic voltammetry (CV) and differential‐capacity (dQ/dV) analyses. The CV curves exhibit distinct and sharp redox peaks at ∼0.32 and ∼0.48 V during delithiation (Figure S10a), indicating highly reversible phase transitions and rapid *Li* ^+^ storage kinetics within the nanoporous architecture. Differential‐capacity (dQ/dV) analysis further clarifies the electrochemical functions of the core–shell architecture (Figure S10b). While the Mg_2_Si and SWCNT@Mg_2_Si electrodes exhibit distinct low‐voltage features in the 0.1–0.2 V region, the nanoporous Si/SiC/SWCNT electrode is dominated by the characteristic Si alloying/dealloying peaks with only a weak residual low‐voltage feature. These observations indicate that the Si‐rich shell serves as the primary *Li*‐storage phase, whereas the retained Mg‐containing Si core contributes only secondarily to *Li* storage while functioning as a mechanically robust, semi‐active backbone that accommodates the overall volume fluctuation of the composite particle.

The interfacial chemistry of the nanoporous Si/SiC/SWCNT electrode was further investigated by XPS before cycling and after the formation cycle (Figure S11). The nanoporous electrode retains a pronounced sp^2^ C─C signal after formation, indicating that the conductive SWCNT network remains structurally preserved during the initial electrochemical reactions. Moreover, the F 1s spectrum is dominated by LiF, whereas the Mg_2_Si electrode exhibits a relatively stronger contribution from Li_x_PF_y_ species originating from LiPF_6_ decomposition. The preferential formation of a LiF‐rich inorganic SEI suggests that the chemically integrated SiC/SWCNT framework promotes a mechanically robust and chemically stable interphase, which contributes to the excellent cycling stability of the nanoporous electrode.

To elucidate the kinetic origin of the enhanced electrochemical performance, electrochemical impedance spectroscopy (EIS) and galvanostatic intermittent titration technique (GITT) measurements were subsequently performed (Figure 4c–e). The Nyquist plots reveal that the nanoporous Si/SiC/SWCNT electrode maintains an exceptionally low charge‐transfer resistance (R_ct_) of only 1.26 Ω even after 30 cycles (Figure 4c), indicating stable interfacial charge transfer throughout prolonged cycling. Consistently, the GITT responses (Figure 4e, Figure S12) exhibit reduced polarization, rapid voltage relaxation, and accelerated lithiation/delithiation kinetics. Accordingly, the calculated *Li* ^+^ diffusion coefficients remain consistently higher than those of all reference electrodes over the entire state‐of‐charge range, spanning from 2.95 × 10^−10^ to 1.29 × 10^−8^ cm^2^ s^−1^. These accelerated ion‐transport kinetics are further reflected in the outstanding rate capability, delivering approximately 1490 mAh g^−1^ even at a high current density of ∼7 A g^−1^ (Figure 4f). These results demonstrate that the hierarchical nanoporous architecture effectively shortens *Li* ^+^ diffusion pathways while maintaining rapid electron transport through the chemically integrated SiC/SWCNT conductive framework.

Finally, the structural origin of the superior cycling stability was directly visualized by cross‐sectional SEM analyses. Before electrochemical cycling, both the pristine Mg_2_Si and nanoporous Si/SiC/SWCNT electrodes exhibit uniform coatings with comparable thickness and intimate adhesion to the Cu current collector (Figure S13a,b). After 30 cycles (Figure S13c,d), the Mg_2_Si electrode already exhibits particle pulverization and partial delamination, whereas the nanoporous Si/SiC/SWCNT electrode largely preserves its original electrode morphology. These structural differences become even more pronounced after 100 cycles (Figure 4g,h). The Mg_2_Si electrode undergoes severe pulverization accompanied by extensive delamination from the Cu current collector, while the nanoporous Si/SiC/SWCNT electrode maintains an integrated electrode structure with minimal thickness variation, as further supported by the corresponding digital photographs. Quantitative thickness analysis further reveals an electrode expansion of approximately 133% for Mg_2_Si but only ∼66% for the nanoporous Si/SiC/SWCNT electrode after 100 cycles. Similar structural degradation is also observed for the intermediate SWCNT@Mg_2_Si and MgO@Si/SiC/SWCNT electrodes (Figure S14), confirming that neither physical SWCNT attachment nor the unetched intermediate structure alone is sufficient to suppress mechanical failure. These structural observations are fully consistent with the electrochemical results, confirming that the synergistic combination of the nanoporous shell, chemically integrated SiC/SWCNT framework, and retained Mg‐containing Si core effectively accommodates repeated volume changes while preserving continuous electron and ion transport pathways throughout prolonged cycling.

### 2.5 Role of the SiC Interfacial Layer in Stabilizing the SWCNT–Si Framework

**FIGURE 5:**
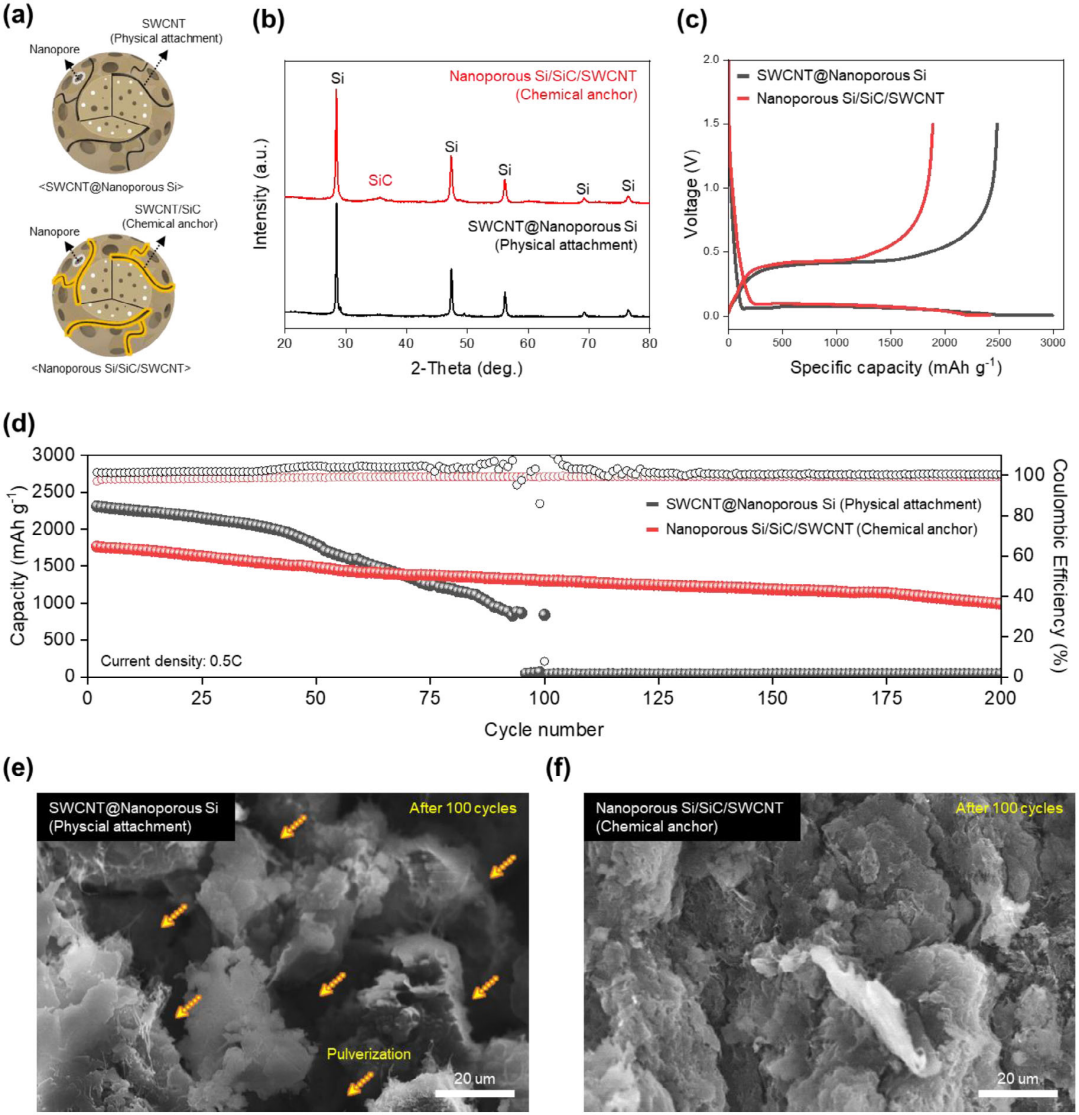
Comparison of physical and chemical anchor of SWCNTs with nanoporous Si. (a) Conceptual schematic illustrating the structural differences between SWCNT@nanoporous Si (physical attachment) and nanoporous Si/SiC/SWCNT (chemical anchor via SiC interface). (b) XRD patterns of the physically attached and chemically integrated samples. (c) Galvanostatic charge/discharge profiles for the two integration strategies. (d) Cycling performance and corresponding Coulombic efficiencies of the electrodes. (e,f) Cross‐sectional SEM images of the SWCNT@Nanoporous Si and nanoporous Si/SiC/SWCNT electrodes, respectively, after 100 electrochemical cycles.

The electrochemical role of the thermochemically generated SiC interfacial layer was directly evaluated by comparing physically attached SWCNT@nanoporous Si with the chemically integrated nanoporous Si/SiC/SWCNT architecture (Figure 5a). In the physically integrated electrode, the SWCNT network is connected to the nanoporous Si surface primarily through weak van der Waals interactions, whereas the chemically integrated counterpart contains an interfacial SiC layer that covalently anchors the SWCNT network onto the nanoporous Si framework. XRD analysis clearly distinguishes these two interfacial configurations (Figure 5b). The chemically integrated nanoporous Si/SiC/SWCNT electrode exhibits distinct β‐SiC diffraction peaks together with crystalline Si, whereas no detectable SiC phase is observed for the physically attached SWCNT@nanoporous Si sample, confirming the absence of chemical interfacial coupling. These differences in interfacial bonding directly govern the electrochemical behavior of the electrodes. As shown in Figure 5c, the physically attached SWCNT@nanoporous Si electrode delivers a slightly higher initial discharge capacity because of the larger fraction of electrochemically active Si and the absence of electrochemically inactive SiC. However, this initial advantage is rapidly lost during prolonged cycling. The physically attached electrode exhibits severe capacity fading together with unstable Coulombic efficiency (Figure 5d), indicating progressive electrical disconnection caused by repeated volume expansion of Si. In contrast, the chemically integrated nanoporous Si/SiC/SWCNT electrode sacrifices only a small fraction of the initial capacity while maintaining substantially improved cycling stability. This structural advantage is directly visualized by post‐cycling cross‐sectional SEM images (Figure 5e,f). After 100 electrochemical cycles, the physically attached SWCNT@nanoporous Si electrode undergoes severe pulverization and structural collapse, whereas the chemically integrated nanoporous Si/SiC/SWCNT electrode largely preserves its original electrode morphology without noticeable structural disintegration. These results clearly demonstrate that the thermochemically generated SiC interfacial layer functions as a robust mechanical and electrical bridge, preserving continuous electron transport while effectively accommodating the repeated volume fluctuations of Si during long‐term cycling.

Although the SiC interfacial layer is indispensable for stabilizing the Si/SWCNT framework, its formation must be carefully optimized. To evaluate the influence of the SiC formation degree, nanoporous Si/SiC/SWCNT electrodes were prepared by thermal treatment at 650°C, 800°C, and 900°C. As shown in Figure 2g,h, the intensity of the SiC diffraction peak gradually increases with increasing treatment temperature, indicating progressive growth of the interfacial SiC layer. Nevertheless, despite the larger amount of SiC formed at elevated temperatures, both the reversible capacity and cycling stability gradually deteriorate (Figure S15a,b). This behavior indicates that excessive SiC formation progressively consumes electrochemically active Si while increasing the fraction of electrochemically inactive SiC [36]. Therefore, the optimized treatment temperature of 650°C provides the best balance between sufficient interfacial stabilization and preservation of active Si, thereby maximizing both reversible capacity and long‐term cycling stability.

Having established the optimum SiC formation condition, the origin of the thermochemically generated SiC interfacial layer was further verified through a control experiment performed under a pure Ar atmosphere without CH_4_. XRD analysis reveals a distinct difference in phase evolution: while the CH_4_‐treated sample predominantly consists of Si and SiC phases, the sample synthesized without CH_4_ retains a substantial amount of Mg_2_Si, particularly near the particle surface (Figure S16a). Notably, although weak SiC peaks are still observable under the CH_4_‐free condition, their relative intensity with respect to Si is significantly lower than that of the CH_4_‐treated counterpart. These observations suggest that solid‐state diffusion of Mg from the Mg_2_Si core toward the surface occurs under both conditions; however, the subsequent extraction and oxidation of Mg are considerably suppressed in the absence of CH_4_. Consequently, the localized exothermic Mg oxidation reaction—which provides thermal activation for Si─C interfacial reactions—is significantly weakened, thereby limiting SiC formation at 650°C. This mechanistic difference is directly reflected in the electrochemical behavior. The CH_4_‐free electrode exhibits a slightly higher initial capacity due to the larger fraction of electrochemically active Si‐containing phases (Figure S16b). However, the absence of a sufficiently developed SiC interfacial framework results in rapid capacity decay during cycling (Figure S16c). In contrast, the CH_4_‐assisted thermochemical process promotes the formation of a robust SiC‐mediated interface, leading to substantially enhanced cycling stability. These findings highlight that the CH_4_‐assisted thermochemical environment is not merely a carbon supply source, but a critical driving factor for constructing the mechanically and electrically stabilized SiC/SWCNT interface.

### 2.6 Pouch‐Type Full‐Cell Demonstration of Nanoporous Si/SiC/SWCNT Anodes

**FIGURE 6:**
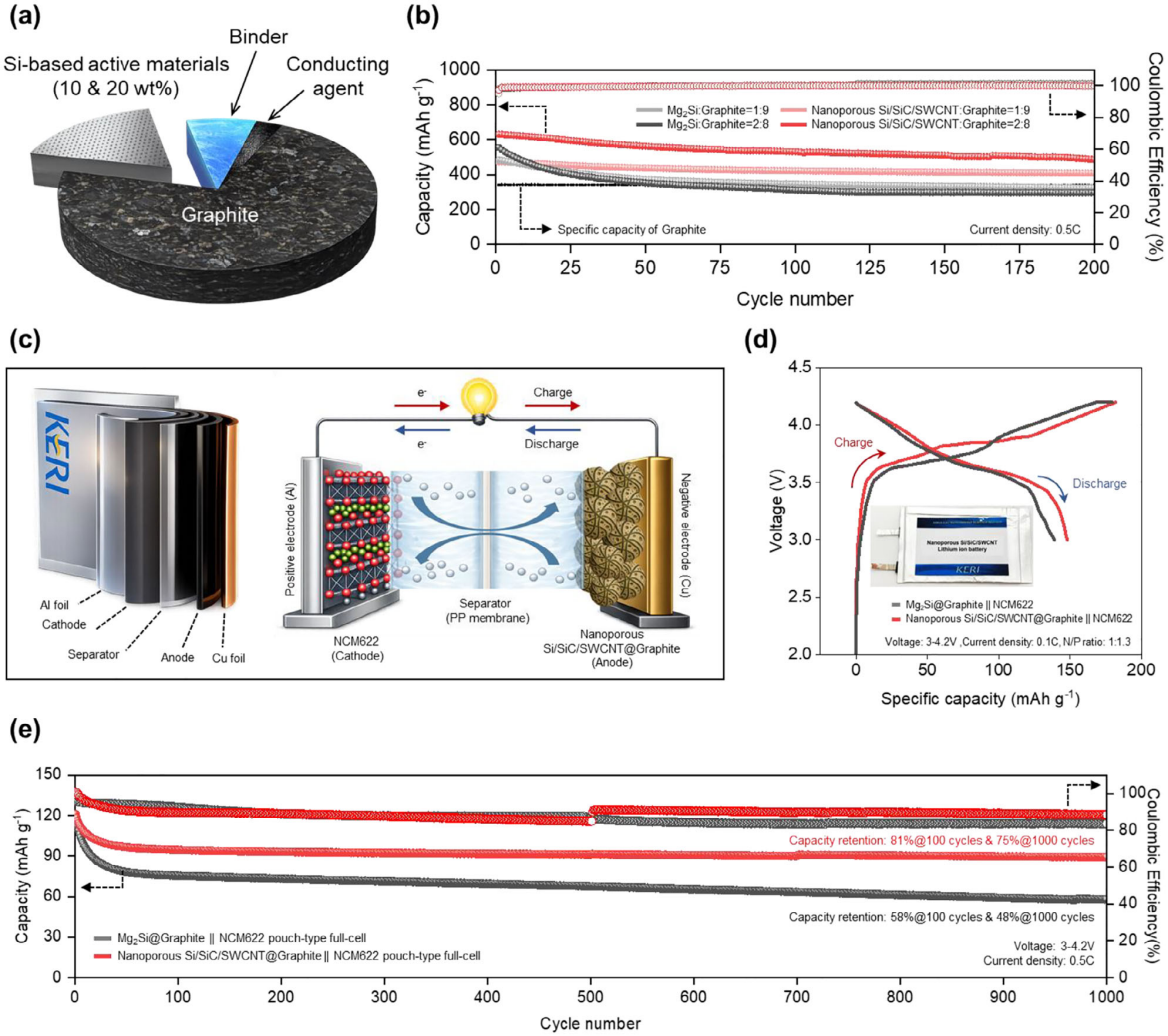
Practical validation of nanoporous Si/SiC/SWCNT‐based anodes in pouch‐type full cells. (a) Schematic diagram of a Si–Graphite composite anode material. (b) Cycling performance and corresponding Coulombic efficiencies of pure graphite, Mg_2_Si:graphite (10:90 and 20:80 wt.%), and nanoporous Si/SiC/SWCNT:graphite (10:90 and 20:80 wt.%) composite electrodes. (c) Schematic illustration of the pouch‐type full‐cell configuration employing an NCM622 cathode and a nanoporous Si/SiC/SWCNT@graphite composite anode. (d) Galvanostatic charge/discharge profiles of Mg_2_Si@graphite || NCM622 and nanoporous Si/SiC/SWCNT@graphite || NCM622 full cells at a constant current density. The inset shows a digital photograph of the assembled pouch‐type full cell. (e) Cycling performance and corresponding Coulombic efficiencies of Mg_2_Si@graphite || NCM622 and nanoporous Si/SiC/SWCNT@graphite || NCM622 full cells.

To evaluate the practical applicability of the nanoporous Si/SiC/SWCNT architecture under realistic electrode conditions, the optimized hybrid was blended with commercial graphite to fabricate Si–graphite composite anodes (Figure 6a). The electrochemical performance of the composite electrodes was systematically compared with those of pure graphite and Mg_2_Si‐based composite electrodes (Figure 6b, Figure S17). When incorporated at a Si‐based composite content of 10 wt.%, the nanoporous Si/SiC/SWCNT@graphite electrode delivered a reversible capacity of 410 mAh g^−1^ after 200 cycles with a retention of 87% at 0.5C. More importantly, even at a significantly increased Si loading of 20 wt.%—substantially exceeding the industrial standard of ∼5 wt.%—the composite electrode maintained a high reversible capacity of 487 mAh g^−1^ with a retention of 78% after 200 cycles. In contrast, the Mg_2_Si:graphite composite electrodes exhibited rapid capacity decay under identical conditions, indicating that the nanoporous structure and chemically stabilized SiC/SWCNT interface play a critical role in suppressing structural degradation at elevated Si contents. These results demonstrate that the engineered core–shell architecture effectively mitigates the intrinsic instability of Si‐based anodes while enabling practical high‐loading Si–graphite electrode designs.

To further validate the practical feasibility of the nanoporous Si/SiC/SWCNT anode under device‐level operating conditions, pouch‐type full cells were assembled using NCM622 cathodes and graphite‐blended anodes (Figure 6c). The full cells were configured with an N/P ratio of 1:1.3 and operated within a voltage range of 3–4.2 V. A formation process was conducted at 0.1 C, followed by long‐term cycling at 0.5C. As shown in Figure 6d, both Mg_2_Si@graphite || NCM622 and nanoporous Si/SiC/SWCNT@graphite || NCM622 full cells exhibit comparable initial discharge capacities of 128 and 130 mAh g^−1^, respectively, suggesting that the initial capacity is primarily governed by the cathode. Nevertheless, the nanoporous Si/SiC/SWCNT‐based full cell exhibits reduced polarization and more stable voltage profiles, indicative of improved reaction kinetics and interfacial stability. More significant differences emerge during prolonged cycling (Figure 6e). The nanoporous Si/SiC/SWCNT@graphite || NCM622 full cell maintains 75% of its initial capacity after 1000 cycles, whereas the Mg2Si@graphite || NCM622 counterpart retains only 48% under identical conditions. In addition, the nanoporous Si/SiC/SWCNT‐based pouch‐type full cell delivers a high energy density of 275 Wh kg^−1^, demonstrating the practical feasibility of the engineered core–shell anode architecture for high‐energy‐density LIBs. The enhanced durability of the nanoporous hybrid‐based full cell can be attributed to the synergistic effects of the engineered core–shell architecture, where the nanoporous Si shell accommodates volumetric expansion, the SiC interfacial layer stabilizes the electrode framework, and the SWCNT conductive network preserves continuous electron transport pathways.

## 3 Conclusion

In this work, we demonstrate a thermochemical core‐shell architecturing for constructing a structurally resilient and kinetically accelerated Si anode through the integration of alloy stabilization, nanoporous strain buffering, and chemically anchored conductive networks. Starting from a SWCNT‐coated Mg_2_Si precursor, the CH_4_‐assisted thermochemical treatment induces localized exothermic Mg oxidation, generating a SiC‐anchored SWCNT conductive network, while subsequent selective etching of MgO produces a nanoporous Si shell surrounding a mechanically robust Mg‐containing Si core. Reactive molecular dynamics simulations further reveal that localized hot‐atom generation during Mg oxidation activates CH_4_ molecules, providing atomistic evidence for the low‐temperature formation of the SiC‐anchored conductive network. This spatially differentiated architecture effectively resolves the long‐standing trade‐off in Si anodes: the dense Mg‐containing Si core mitigates absolute volume fluctuations, while the nanoporous SiC/SWCNT shell provides strain accommodation, fast *Li* ^+^ transport pathways, and stable electronic percolation. Consequently, the hybrid electrode exhibits reduced charge‐transfer resistance, accelerated *Li* ^+^ diffusion kinetics, and significantly enhanced cycling stability compared with conventional Si‐based systems.

Importantly, the advantages of this architecture extend to practical electrode configurations. Even at a high Si content of 20 wt.% in graphite composite electrodes—substantially exceeding the typical industrial level (∼5 wt.%)—the material maintains stable cycling performance, highlighting the effectiveness of the integrated structural design in managing severe volume changes. Furthermore, pouch‐type full‐cell evaluations paired with NCM622 cathodes confirm the practical viability of the system under realistic cell configurations. These findings establish thermochemical structural engineering as a scalable route for constructing multifunctional Si‐based architectures and offer a promising strategy for developing durable, high‐capacity anodes for next‐generation LIBs.

## 4 Experimental Section

### 4.1 Materials

A SWCNT dispersion (0.4 wt.% single‐walled carbon nanotubes, 0.6 wt.% dispersing agent, and 99.0 wt.% water; TUBALL) was purchased from OCSiAl Asia Pacific Co., Ltd. Silicon powder (99%, trace metals basis) and magnesium powder (99%) were obtained from Sigma–Aldrich. All chemicals were used as received without further purification.

### 4.2 Synthesis of Nanoporous Si/SiC/SWCNT

Mg_2_Si alloy was synthesized via a HEBM process. Si and Mg powders were mixed at a molar ratio of Si:Mg = 55:45, with a total mass of 3 g. The mixture was mechanically alloyed for 6 h under an Ar atmosphere to induce the formation of the Mg_2_Si phase. To prepare the SWCNT@Mg_2_Si hybrid, SWCNTs were introduced onto the surface of Mg_2_Si particles. Mg_2_Si powder and SWCNTs were mixed at a weight ratio of 80:1 and dispersed in deionized water (1 g per 100 mL). The suspension was subsequently processed via spray drying, resulting in uniform coating of SWCNTs on the Mg_2_Si particles. The SWCNT@Mg_2_Si hybrid was further subjected to a CVD process. The heat treatment was conducted in a tubular furnace under a CH_4_/Ar atmosphere (CH_4_: 200 sccm, Ar: 800 sccm). The temperature was increased from room temperature to 650°C at a heating rate of 10°C min^−1^ and maintained for 3 h, followed by natural cooling to room temperature under an Ar atmosphere. The resulting MgO@Si/SiC/SWCNT was then treated in a diluted hydrochloric acid solution (1 m HCl) to remove the MgO phase. The composite powder was dispersed in the solution (1 g per 100 mL) and stirred for 2 h, followed by centrifugation. The collected precipitate was repeatedly washed with deionized water until the pH reached approximately neutral. Finally, the product was dried in a vacuum oven for 8 h to obtain the nanoporous Si/SiC/SWCNT.

### 4.3 Synthesis of SWCNT@nanoporous Si

Mg_2_Si precursor was first synthesized via the same HEBM process described above. The obtained Mg_2_Si powder was subsequently subjected to the same thermochemical treatment and acid‐etching procedure as described for the preparation of nanoporous Si/SiC/SWCNT, except that no SWCNTs were introduced prior to the heat treatment, yielding nanoporous Si. To prepare the SWCNT@nanoporous Si control sample, the nanoporous Si powder and SWCNTs were mixed at a weight ratio of 80:1 and dispersed in deionized water (1 g per 100 mL). The suspension was then processed via the same spray‐drying procedure described above, followed by vacuum drying for 8 h to obtain the SWCNT@nanoporous Si sample.

### 4.4 Reactive Molecular Dynamics Simulations

The rMD simulations were performed with LAMMPS integrated with the Reaxff package [37, 38]. A heterogeneous bilayer model was constructed by periodically arranging alternating Mg_2_Si alloy and crystalline Si domains with lateral dimensions of 2 nm and a thickness of 2 nm for each domain. Periodic boundary conditions were applied in the in‐plane directions (x and y), while a 3 nm thick vacuum region was introduced along the surface normal (z direction) to accommodate the gas‐phase species. Prior to atmospheric exposure, the bilayer structure was equilibrated at 300 K under an in‐plane pressure of 1 atm for 1 ns using the NPT ensemble, where pressure coupling was applied only along the lateral directions while maintaining the vacuum thickness along the surface normal. Following structural equilibration, the surrounding atmosphere was represented by introducing O_2_ and CH_4_ molecules at a molar ratio of 1:1 into the vacuum region. Gas molecules were randomly inserted into the vacuum space at a rate of one molecule every 1 ps to emulate continuous atmospheric exposure during polymer decomposition. The subsequent reactive molecular dynamics simulations were carried out for 1 ns using a time step of 0.1 fs.

### 4.5 Material Characterization

The morphology and microstructure of the samples were characterized using field‐emission SEM (S4800, Hitachi, Japan). Elemental distributions were analyzed by EDS mapping coupled with TEM (Titan G2 60–300, FEI, USA). High‐resolution TEM was employed to investigate the internal structure and confirm the formation of SiC phases. The crystal structure and phase composition were analyzed using XRD with Cu Kα radiation (λ = 1.5406 Å) over a 2θ range of 10°–80°. Raman spectroscopy (excitation wavelength: 633 nm, RENISHAW, South Korea) was used to examine the structural characteristics of carbon and silicon components. Surface chemical composition and bonding states were analyzed using XPS (Multilab 2000, Thermo VG Scientific Inc., USA).

### 4.6 Electrochemical Measurements

Working electrodes were prepared by mixing the Si‐based active materials (Mg_2_Si, SWCNT@Mg_2_Si, MgO@Si/SiC/SWCNT, and nanoporous Si/SiC/SWCNT), carbon black, and polyacrylic acid (PAA) binder at a weight ratio of 80:10:10. The mixture was dispersed in deionized water using a planetary centrifugal mixer (Thinky Mixer, ARE‐310) to form a homogeneous slurry. The slurry was coated onto Cu foil (10 µm thickness) using a doctor blade, followed by drying at 80 °C for 5 min and subsequent vacuum drying at room temperature for 12 h. The mass loading of the active material was approximately 3–3.3 mg cm^−2^. For graphite‐blended electrodes, the nanoporous Si/SiC/SWCNT composite and graphite were used as active materials with Si‐based composite contents of 10 and 20 wt.%. The active materials, carbon black, and sodium carboxymethyl cellulose/styrene‐butadiene rubber (S‐CMC/SBR) binder were mixed at a weight ratio of 91:3:6. The slurry preparation and electrode fabrication procedures were identical to those described above. All electrodes were fabricated using identical slurry compositions, binder/carbon contents, active material loadings, coating procedures, and drying conditions. The electrical properties of the fabricated electrode sheets were evaluated using a Hioki RM2610 Electrode Resistance Measurement System (Hioki, Japan), consisting of an RM2611 Electrode Resistance Meter, RM2612 Resistance Calculation Software, RM9003 Press Unit, and RM9004 Test Fixture. The coated surface of each electrode was directly contacted by a multiprobe test fixture containing 46 needle‐type probes arranged in a grid with an approximately 120 µm probe pitch. The composite‐layer thickness, Cu current collector thickness, and Cu current collector volume resistivity were used as input parameters for the resistance calculation. The composite‐layer volume resistivity (Ω·cm) and the area‐specific interface resistance (Ω·cm^2^) between the composite layer and Cu current collector were determined by fitting the calculated potential distribution to the experimentally measured potential distribution using the dedicated analysis software. Circular electrodes (1.54 cm^2^) were punched and assembled into CR2032 coin cells in an Ar‐filled glove box using Li metal as both the counter and reference electrodes. A polypropylene separator (Celgard 2400) and an electrolyte consisting of 1 M LiPF_6_ dissolved in ethylene carbonate (EC)/diethyl carbonate (DEC) with fluoroethylene carbonate (FEC) additive were used. Pouch‐type full cells were assembled in an Ar‐filled glove box using graphite‐blended nanoporous Si/SiC/SWCNT composite anodes containing 20 wt.% Si‐based composite and commercially available NCM622 cathodes in a stacked configuration separated by a polypropylene separator (Celgard 2400). The graphite‐blended anode consisted of 20 wt% nanoporous Si/SiC/SWCNT composite and 80 wt.% graphite, with carbon black and sodium carboxymethyl cellulose/styrene‐butadiene rubber (S‐CMC/SBR) binder at a weight ratio of 91:3:6. The areal mass loading of the anode and cathode was approximately 3.3 and 3 mg cm^−2^, respectively, corresponding to an N/P ratio of approximately 1:1.3. Aluminum and nickel tabs were welded onto the cathode and anode current collectors, respectively. The electrolyte consisted of 1 m LiPF_6_ dissolved in EC/DEC with FEC additive, and approximately 1000 µL of electrolyte was injected under vacuum to ensure sufficient wetting of the electrodes. The electrode stack was then enclosed in an aluminum‐laminated pouch film and vacuum‐sealed. Prior to long‐term cycling, the pouch cells were subjected to two formation cycles at 0.1C before electrochemical evaluation. Galvanostatic charge/discharge measurements were performed within a voltage window of 3.0–4.2 V at a current density of 0.5C. CV measurements were performed using a VMP3 electrochemical workstation within a voltage window of 0–1.5 V. Galvanostatic charge/discharge measurements were conducted using a battery tester (WonA Tech, Korea). EIS measurements were carried out over a frequency range from 1 MHz to 0.01 Hz with an amplitude of 10 mV. *Li* ^+^ diffusion kinetics were evaluated using the GITT at 0.1C with a pulse duration of 10 min. CV measurements for pouch‐type full cells were performed within a voltage window of 3.0–4.2 V.

## Author Contributions

**Dong Gyun Hong**: methodology, data curation, investigation, validation, formal analysis. **Byeong Guk Kim**: methodology, investigation. **Jung‐Hyeok Park**: methodology, investigation. **Sunwoo Lee**: methodology, investigation. **Donghwi Cho**: investigation, methodology. **Jaeik Hyun**: methodology, investigation. **Ki‐Hun Nam**: methodology, investigation. **Youngoh Kim**: investigation, data curation, validation. **Geon‐Woong Lee**: methodology, investigation. **Sunhye Yang**: methodology, investigation, funding acquisition, project administration. **Seung Yol Jeong**: methodology, investigation, project administration. **Myungwoo Choi**: conceptualization, methodology, data curation, investigation, validation, formal analysis, supervision, visualization, writing – original draft, writing – review and editing.

## Conflicts of Interest

The authors declare no competing interests.

## Supporting information




**Supporting File**: advs77441‐sup‐0001‐SuppMat.docx.

## Data Availability

The authors declare that all data supporting the findings of this study are available in the article and its Supporting Information file. Further data sets are available from the corresponding author upon reasonable request.
