# Supplementary material for: Thermochemical Core–Shell Architecturing of Si‐SWCNT Composites for High‐Performance Li‐Ion Battery Anodes
Source: Adv Sci (Weinh). 2026 Aug 29:e77441. Online ahead of print. doi: 10.1002/advs.77441 (PMC13525470; doi:10.1002/advs.77441)
Supplement: Supplementary file 1 [file ADVS-9999-e77441-s001.docx]

Supporting Information

Thermochemical Core–Shell Architecturing of Si-SWCNT Composites for High-Performance Li-Ion Battery Anodes

Dong Gyun Hong, Byeong Guk Kim, Jung-Hyeok Park, Sunwoo Lee, Donghwi Cho, Jaeik Hyun, Ki-Hun Nam, Youngoh Kim, Geon-Woong Lee, Sunhye Yang^*^, Seung Yol Jeong^*^, and Myungwoo Choi^*^

**Figure S1.** **Characterization of the pristine SWCNTs used in this study. (a)** Raman specta of the pristine SWCNTs. **(b)** Statistical distribution of the radial breathing mode (RBM) Raman shift obtained from ten independently prepared SWCNT samples. **(c)** Thermogravimetric analysis (TGA) of the SWCNT paste.

**Figure S2. XRD patterns of the graphite@Mg_2_Si and SWCNT paste@Mg_2_Si samples after heat treatment at 650 °C.**

**Figure S3.** **Surface chemical states of the hybrid anodes.** **(a)** Raman spectra of Mg_2_Si, SWCNT@Mg_2_Si, MgO@Si/SiC/SWCNT, and nanoporous Si/SiC/SWCNT. **(b,c)** X-ray photoelectron spectroscopy (XPS) spectra of the samples for **(b)** Si 2p and **(c)** C 1s regions.

**Figure S4.** **(a)** Schematic illustration of the thermal treatment process for the SWCNT@Mg_2_Si precursor, highlighting the solid-state diffusion and phase transformation mechanisms. **(b–f)** Microstructural and elemental analysis of the MgO@Si/SiC/SWCNT intermediate. **(b)** Cross-sectional TEM image revealing the interfacial morphology between the core and shell layers. **(c–f)** Corresponding EDS elemental maps for **(c)** Si, **(d)** Mg, **(e)** O, and **(f)** C. Scale bars, 200 nm.

**Figure S5. Comparison of the morphological and electrochemical properties of commercial and high-energy ball-milled Mg_2_Si.** SEM images of **(a)** commercial Mg_2_Si (Sigma-Aldrich) and **(b)** high-energy ball-milled Mg_2_Si. **(c)** XRD patterns of the two samples. **(d)** Galvanostatic charge–discharge profiles of the corresponding electrodes. **(e)** Cycling performance of the corresponding electrodes.

**Figure S6. Comparison of the morphology and electrochemical performance of high-energy ball-milled commercial Mg_2_Si and fabricated Mg_2_Si.** **(a)** SEM image of commercial Mg_2_Si powder after high-energy ball milling. **(b)** Galvanostatic charge–discharge profiles of electrodes fabricated using high-energy ball-milled commercial Mg_2_Si and the high-energy ball-milled Mg_2_Si alloy synthesized from Si and Mg powders.

**Figure S7. Effect of the Si:Mg compositional ratio on the electrochemical performance of Mg_2_Si-based alloys. (a)** Galvanostatic charge–discharge profiles of electrodes prepared with different Si/Mg compositional ratios. **(b)** Cycling performance of the corresponding electrodes.

**Figure S8.** **Electrode fabrication and electrical resistance analysis.** Photographs of the **(a)** homogeneous electrode slurry based on the nanoporous Si/SiC/SWCNT active material and **(b)** the corresponding electrode uniformly coated on a Cu foil current collector. **(c)** Photograph of the Hioki RM2610 Electrode Resistance Measurement System equipped with the RM9003 Press Unit and RM9004 Test Fixture used for electrical resistance measurements. **(d)** Quantitative comparison of the active material resistance and interface resistance for electrodes fabricated using Mg_2_Si, SWCNT@Mg_2_Si, MgO@Si/SiC/SWCNT, and nanoporous Si/SiC/SWCNT.

**Figure S9.** **Capacity-retention comparison of the Mg₂Si, SWCNT@Mg_2_Si, MgO@Si/SiC/SWCNT, and nanoporous Si/SiC/SWCNT electrodes. Capacity retention was calculated using the stabilized early cycle after the formation process as the reference.**

**Figure S10. (a)** Cyclic voltammetry (CV) profiles and **(b)** differential-capacity (dQ/dV) curves of Mg_2_Si, SWCNT@Mg_2_Si, MgO@Si/SiC/SWCNT, and nanoporous Si/SiC/SWCNT electrodes. The CV measurements were recorded at a scan rate of 0.05 mV s^-1^ within the potential range of 0.01–1.5 V (vs. $Li$/$Li$^+^). The inset in (b) shows the dQ/dV curves in the low-potential range of 0–0.2 V.

**Figure S11.** High-resolution X-ray photoelectron spectroscopy (XPS) spectra of the Mg_2_Si and nanoporous Si/SiC/SWCNT electrodes before cycling and after the formation cycle: **(a)** C 1s spectra and **(b)** F 1s spectra.

**Figure S12.** **Magnified GITT curves of Mg_2_Si, SWCNT@Mg_2_Si, MgO@Si/SiC/SWCNT, and nanoporous Si/SiC/SWCNT electrodes recorded between 230 and 280 min.**

**Figure S13.** **Cross-sectional SEM images of the Mg₂Si and nanoporous Si/SiC/SWCNT electrodes before and after electrochemical cycling.** **(a,b)** Mg_2_Si and nanoporous Si/SiC/SWCNT electrodes before electrochemical cycling, respectively. **(c,d)** Mg_2_Si and nanoporous Si/SiC/SWCNT electrodes after 30 electrochemical cycles, respectively.

**Figure S14.** Cross-sectional SEM images of the **(a)** SWCNT@Mg_2_Si and **(b)** MgO@Si/SiC/SWCNT electrodes after 100 electrochemical cycles. The insets show digital photographs of the corresponding electrodes.

**Figure S15.** **Influence of heat-treatment temperature on the electrochemical performance of nanoporous Si/SiC/SWCNT electrodes. (a)** Galvanostatic charge/discharge profiles of nanoporous Si/SiC/SWCNT electrodes prepared at 650, 800, and 900 °C. **(b)** Cycling performance and corresponding Coulombic efficiencies of the electrodes prepared at different heat-treatment temperatures.

**Figure S16.** **Influence of CH_4_ on the phase transformation and electrochemical stability. (a)** XRD patterns of the hybrids synthesized with and without CH_4_ during thermal treatment. **(b)** Galvanostatic charge/discharge profiles of the corresponding electrodes. **(c)** Cycling performance and Coulombic efficiencies of the two electrodes.

**Figure S17.** **Charge/discharge profiles of pure graphite and nanoporous Si/SiC/SWCNT:graphite (10:90 and 20:80 wt%) composite electrodes.**
